# Preparation and characterization of lignin-derived carbon aerogels

**DOI:** 10.3389/fchem.2023.1326454

**Published:** 2024-01-08

**Authors:** Piia Jõul, Oliver Järvik, Heidi Lees, Urve Kallavus, Mihkel Koel, Tiit Lukk

**Affiliations:** ^1^ Department of Chemistry and Biotechnology, Tallinn University of Technology, Tallinn, Estonia; ^2^ Department of Energy Technology, Tallinn University of Technology, Tallinn, Estonia; ^3^ Department of Mechanical and Industrial Engineering, Tallinn University of Technology, Tallinn, Estonia

**Keywords:** aerogels, carbon aerogels, lignin, resorcinol–formaldehyde gels, supercritical drying, pyrolysis

## Abstract

Lignin is considered a valuable renewable resource for building new chemicals and materials, particularly resins and polymers. The aromatic nature of lignin suggests a synthetic route for synthesizing organic aerogels (AGs) similar to the aqueous polycondensation of resorcinol with formaldehyde (FA). The structure and reactivity of lignin largely depend on the severity of the isolation method used, which challenges the development of new organic and carbon materials. Resorcinol aerogels are considered a source of porous carbon material, while lignin-based aerogels also possess great potential for the development of carbon materials, having a high carbon yield with a high specific surface area and microporosity. In the present study, the birch hydrolysis lignin and organosolv lignin extracted from pine were used to prepare AGs with formaldehyde, with the addition of 5-methylresorcinol in the range of 75%–25%, yielding monolithic mesoporous aerogels with a relatively high specific surface area of up to 343.4 m^2^/g. The obtained lignin-based AGs were further used as raw materials for the preparation of porous carbon aerogels (CAs) under well-controlled pyrolysis conditions with the morphology, especially porosity and the specific surface area, being dependent on the origin of lignin and its content in the starting material.

## Introduction

For many applications, it is important to have a porous material with a well-defined and uniform structure with a specific surface area, pore structure, and pore size that can be tuned according to the needs of a particular application; in such cases, both organic and carbon aerogels (AGs and CAs) possess very good potential to provide this kind of materials (Estevez et al., 2017; Qiang et al., 2017; Li et al., 2019). Most carbon products are obtained by heating coal (to give coke), natural gas (to give carbon black), or other carbonaceous materials of plant or animal origin (to give charcoal at elevated temperatures). Nowadays, more attention is being paid to using biomass as a source of porous materials not because of using renewable raw materials but because it can provide more diverse porous materials with tuneable properties in an economical way.

Lignin comprises 10%–25% of the total renewable lignocellulosic biomass, and it is the most abundant natural source of aromatics (Ragauskas et al., 2014; Dessbesell et al., 2020). Three primary phenylpropanoid monomer units, namely, syringyl-(S), guaiacyl-(G), and *p*-coumaryl-(H), constitute the basis for providing a good starting platform for a wide range of chemicals (Panga et al., 2017; Suota et al., 2021). The effective extraction or separation of lignin with a stable structure and high purity from biomass is an important step for further valorization. For that, the bond rupture to separate lignin from carbohydrates and its partial depolymerization to make lignin extractable are required, resulting in its solubilization in a proper solvent for further processing (Sun et al., 2018).

Lignin is largely available from the papermaking industry processes like kraft pulping, sulfite pulping, and soda pulping. Other methods, such as hydrolysis/fractionation using hot water, dilute acid, and alkaline and organic solvents, are being utilized on a smaller scale (Zakzeski et al., 2010; Zhao and Abu-Omar, 2021). In active research, the focus is on using enzymes and developing biorefinery processes (Bornscheuer et al., 2014; Fabbri et al., 2023). For environmentally friendlier processes, soda pulping and organosolv (OS) extraction can be proposed, where the lignin obtained is sulfur-free. However, in all these areas, lignin is considered a waste with a very low industrial value, except as fuel for particular industries.

The phenolic nature of lignins makes them potential replacements for phenol/resorcinol in a multitude of industrial applications. A synthetic route of producing AGs by the aqueous polycondensation of resorcinol (1,3-dihydroxybenzene) with formaldehyde (FA) was proposed by Pekala (1989). When lignin contains a free phenolic hydroxyl group and abundant vacant *ortho*- or *para*-sites, it can react with FA to yield polymeric gels. The first attempts at making lignin–phenol–formaldehyde resins were already reported 20 years ago (Effendi et al., 2008; Chen et al., 2011; Grishechko et al., 2013). Various drying processes, such as drying with supercritical CO_2_, drying in ambient conditions, and freeze-drying, were used, producing AGs, xerogels, and cryogels, respectively.

The different amounts of the primary phenylpropanoid monomer units in lignin provide different lignin reactivities. It should be noted that H-type units possess more than one active site, offering the potential to create highly cross-linked structures. In the G-type units, one *ortho-*position is already occupied by a methoxyl group, whereas in the S-type units, both are occupied. The H- and G-units are able to react with FA to synthesize lignin-based resins, while the S-unit (no active site is present) is not. In this regard, softwood lignins, which contain mainly G-units, are a better choice for lignin-based resin synthesis than hardwood lignins, which contain S- and some H-units. Spectroscopic methods like infrared spectroscopy (IR) and nuclear magnetic resonance (NMR) are important means that characterize the particular lignin structure prior to material development (Brudin and Schoenmakers, 2010; Singh et al., 2023).

In addition to the different origins, the structure and reactivity of lignins largely depend on the severity of the isolation method used, which must be considered for any desired application. This is one of the greatest challenges for the development of new materials (Titton Dias et al., 2017; Boarino and Klok, 2023). Alkali and AG lignins with a higher number of G-units, free *ortho-*position, and moderate molecular weight are better candidates for the synthesis of 100% lignin-based resins (Raj et al., 2020). Therefore, due to the nature of the reaction between lignin and FA, the structure of lignin is more important than its molecular weight and polydispersity in the synthesis of lignin-based AGs. Although lignins are less reactive toward the addition/substitution reactions due to the lack of reactive sites, the replacement of phenol with lignin would be economically highly advantageous as the phenolic resins are used in many industrial applications such as in the automotive field, computing, aerospace, and construction, as well in the manufacture of engineered wood products (Upton and Kasko, 2016; Gao et al., 2021).

In different developments, phenolic resins and resorcinol AGs are considered useful sources for porous carbon materials, having some distinct advantages such as high carbon yield and high microporosity of the carbonized materials, even without any activation (Jõul et al., 2018; Li et al., 2019; Jõul et al., 2021). The same tendency has also been observed for lignin-derived materials (Xua et al., 2018; Barra et al., 2022; Wu et al., 2022). Thermal treatment and carbonization of lignin-based resins are attractive topics for the research on porous carbons and carbon fibers (Ren et al., 2021). The thermal degradation of lignin is a complex process because of the variety of structural units with different decomposition pathways, including competitive and/or consecutive reactions. The thermal decomposition process of lignin in the temperature range of 20°C–900°C is divided into several distinctive steps with different degradation products and complex changes in the material structure (Talabia et al., 2020; Huang et al., 2022). It is generally believed that the pyrolysis of lignin mainly occurs via free radical reactions (Ma et al., 2023). In the molecular structure of lignin, the oxygen bridge bonds connecting phenyl-propane units and side chains are easily broken when heated. Active free radicals containing benzene rings are formed, and these can easily react with other molecules or free radicals to generate macromolecules with more stable structures, eventually forming biochar at higher temperatures (Chen et al., 2022). These conclusions were drawn from studies on thermal properties, including thermal stability, and thermal degradation kinetics of lignin (Wang et al., 2022). The pyrolysis process of lignin is dominated by the condensation reaction and degradation and rearranging of smaller structural units within it. In the third stage of lignin pyrolysis above 500°C, the aromatic ring-opening, condensation reactions and the formation of char occur (Ren et al., 2021). With the increasing pyrolysis temperature, the rapid decomposition of the biomass and the release of volatiles lead to the formation of pores in the resulting biochar (Chen et al., 2022). There are discussions about the direct use of biomass for carbon materials, and they also point toward the high variability and complicated control of pyrolysis of the material (Matveeva and Bronstein, 2022). Different studies indicate that pyrolysis conditions have a notable impact on the carbon material morphology, and high heating rates lead to the plastic deformation of particles (i.e., melting), resulting in smooth surfaces and large cavities (Cetin et al., 2005; Choi et al., 2022). It has been reported that the porosity and high surface area of lignin-based CAs are generated by the simultaneous pyrolysis and KOH activation of lignin-based organic AGs (Xua et al., 2018). Therefore, a careful study of the structural components of lignin, its various sources, surface area, pore size, and pore distribution is essential for understanding the post-pyrolysis behavior of the obtained carbon materials (Mehta et al., 2020).

Considering the above-mentioned aspects of lignins, including their chemical nature and the resulting behavior, the aim of this work is to continue the studies on the synthesis of lignin-based AGs using different raw materials and the use of these organic porous materials to develop a reliable process for porous CA formation, including a thorough characterization of the materials obtained.

## Materials and methods

### Chemicals

Ethanol, dioxane, hydrochloric acid, acetic acid, sodium hydroxide, sulfuric acid, the FA solution (37 wt% in H_2_O), and the sodium carbonate powder (≥99.0%) were purchased from Sigma-Aldrich (Germany). All the chemicals were of analytical grade and were used as received. Deionized water from a Milli-Q water purification system (Millipore S.A.S., Molsheim, France) was used throughout the study. 5-Methylresorcinol (5-MR) with a reported purity of >99% was provided by AS VKG (Estonia).

### Raw materials and the methodology of the preparation of aerogel

Two types of lignin were used in this project: hydrolysis lignin from birch trees and OS lignin obtained from pine trees. Fibenol OÜ (Estonia) provided the birch hydrolysis lignin. Pine timber sawdust for OS lignin was provided by the Laboratory of Wood Technology (Tallinn University of Technology). All the raw materials were dried in a convection oven at 50°C to 8% moisture, followed by grinding to a fine powder and storing in plastic bags at room temperature. The AG isolation of lignin with ethanol and dioxane from this biomass was performed according to the process described in Jõul et al. (2022).

**FIGURE 1:**
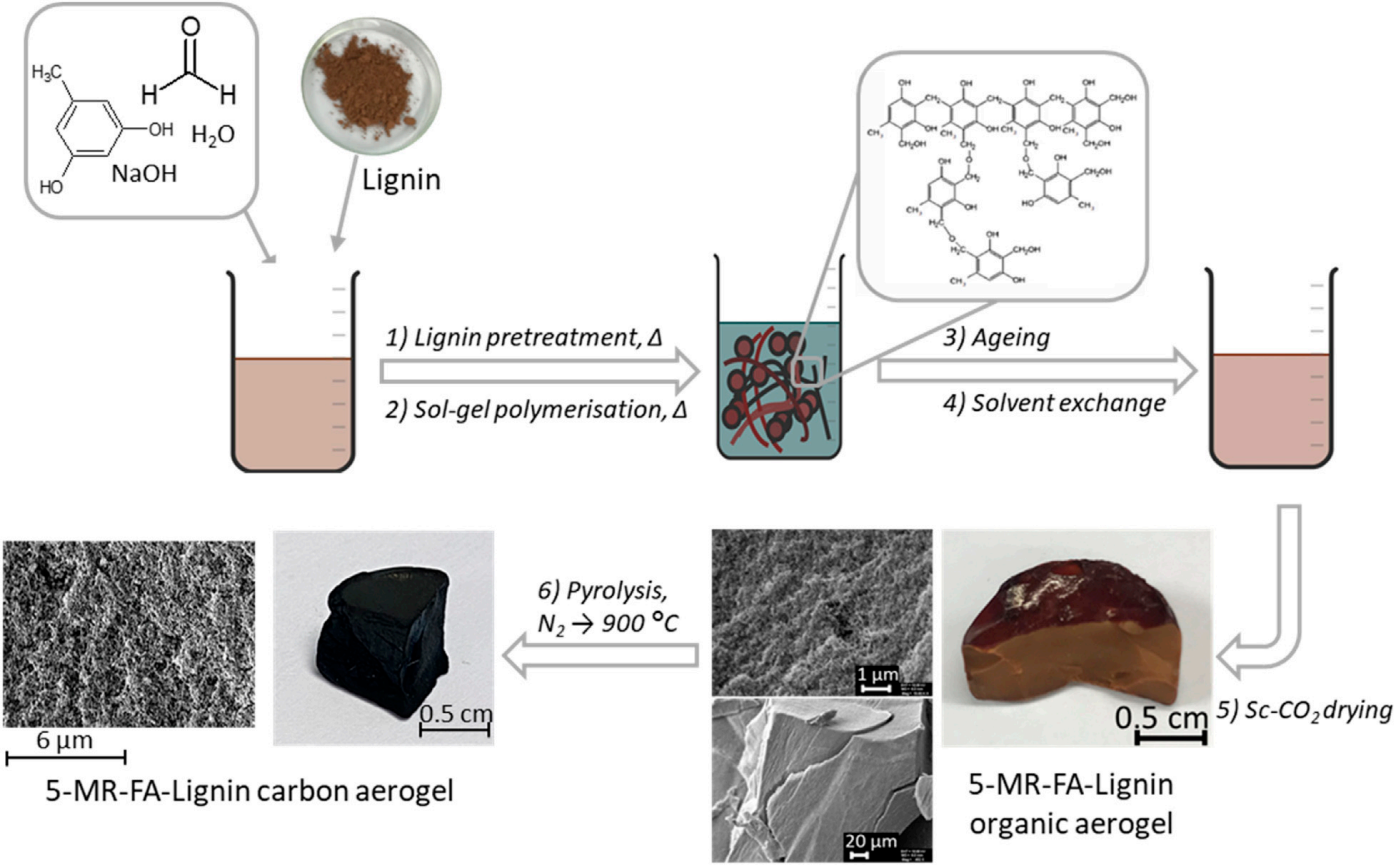
Process of CA preparation.

The lignins were used to prepare the lignin-5-MR-FA AGs via the sol–gel polycondensation process, according to the procedure described in a previous paper by Jõul et al. (2022). The process is shown in Figure 1. Shortly, the procedure started with lignin homogenization in ultrapure water at 85°C with 0.09% NaOH (wt% based on lignin), and the pH of the mixture was close to 10. After that, the water solution of 5-MR and FA was added to the cooled mixture of lignin and homogenized by mixing on a vortex. The reaction mixtures were chosen so that 25%–75% of 5-MR was replaced with a particular type of lignin. The mixture was kept at 85°C to allow gelation for 1–3 h. The gels were let to age in 1% acetic acid for 12 h. Before supercritical drying, the solvent was exchanged for acetone. The gels were dried by dynamic supercritical CO_2_ drying (100 bar, 1.5 h, 25°C; 120 bar, 2.5 h, 45°C). For this purpose, a supercritical extraction system—a 100-mL double-clamp autoclave (NWA Analytische Meßgeräte GmbH, Germany)—was used. Samples under the study are listed in Table 1.

**TABLE 1:** Samples under study and the parameters of the prepared AGs.

Organic aerogel (AG)		Carbon aerogel (CA)	Comment
	Amount of 5-MR replaced by lignin, %	Carbonization—25°C–950°C, N_2_	Precursors, lignin type
AG-5-MR-FA	0	CA-5-MR-FA	Pure 5-MR-FA
AG-HL-75	75	CA-HL-75	Hydrolysis lignin, birch
AG-HL-50	50	CA-HL-50	Hydrolysis lignin, birch
AG-HL-25	25	CA-HL-25	Hydrolysis lignin, birch
AG-EOL-Pine	75	CA-EOL-Pine	Ethanol organosolv lignin, pine
AG-DOL-Pine	75	CA-DOL-Pine	Dioxane organosolv lignin, pine

AGs and CAs were analyzed for morphological characteristics and thermal decomposition.

To obtain CAs, pyrolysis was carried out in the N_2_ atmosphere using an MTF 12/38/400 pyrolysis oven (Carbolite Gero, England). The optimization of the pyrolysis temperature program for conducting carbonization was performed using different process steps and heating rates. Finally, throughout the research, the following temperature program was applied: from 25°C to 300°C at a rate of 10°C/min (held for 10 min), 300°C–550°C at 10°C/min (held for 10 min), and finally, increased to 900°C at 10°C/min (held for 60 min). After pyrolysis, the furnace was allowed to cool to room temperature in the N_2_ atmosphere.

### Characterization of the materials’ morphology

The pore structures of the AG samples were investigated by the N_2_ adsorption–desorption method at 77 K with the QuantaChrome autosorb iQ apparatus in the relative pressure range of 0.005–0.995. Prior to analysis, the AG powder was degassed at 105°C for 24 h to remove surface impurities and moisture. The specific surface area (S_BET_) was calculated by applying the Brunauer–Emmett–Teller (BET) method, and the total pore volume was determined by the volume of N_2_ adsorbed at a relative pressure of 0.99. The pore size distribution was determined using the density functional theory (DFT). The data are presented in Table 2.

**TABLE 2:** Structural data on the studied AGs.

Organic aerogel (AG)				Carbon aerogel (CA)			
	S_BET_, m^2^/g	Total pore volume, cm^3^/g^a^	Average pore diameter, nm		S_BET_, m^2^/g	Total pore volume, cm^3^/g^a^	Average pore diameter, nm
AG-5-MR-FA	224.3	1.72	30.6	CA-5-MR-FA	501.2	0.36	2.85
AG-HL-75	299.5	1.39	18.6	CA-HL-75	108.9	0.24	8.89
AG-HL-50	343.4	1.04	20.7	CA-HL-50	254.3	0.36	17.0
AG-HL-25	244.6	0.62	16.0	CA-HL-25	434.9	0.65	18.3
AG-EOL-Pine	424.1	0.94	8.9	CA-EOL-Pine	20.9	0.04	7.36
AG-DOL-Pine	393.2	1.13	11.47	CA-DOL-Pine	345.7	0.29	3.36

^a^ Total pore volume at P/P_0_ = 0.99 (cm3/g).

Scanning electron microscopic (SEM) images were generated to characterize the morphology of the resulting AGs. The surface morphology of both AGs and CAs was examined with a high-resolution scanning electron microscope Zeiss EVO MA 15 SEM at an accelerating voltage of 10 kV. For imaging, pieces of AG samples were fractured into smaller parts to open the internal structure and view the particles’ close morphology. The fractured pieces were attached with a double-sided adhesive tape to the stub and coated with the Ag/Pd conductive layer in the Fine Coat Ion Sputter JFC-1100.

The ground samples of AGs were spotted on a diamond crystal and analyzed on an IRTracer-100 FTIR spectrophotometer (Shimadzu, Japan) in the attenuated total reflection (ATR) mode. The spectra were recorded over the 750–4,000 cm^−1^ range by averaging 20 scans at a maximum resolution of 2 cm^−1^ and analyzed using LabSolutions software (Thermo Fisher Scientific).

The micro-Raman spectra were recorded using a Horiba LabRam HR800 spectrometer and a 532-nm Nd-YAG laser focused on the sample with a spot size of approximately 5 μm.

### Thermal analysis of materials

The thermogravimetric analysis (TGA) of the AG samples was performed using a NETZSCH STA 449 *F3* Jupiter^®^ thermal analyzer. For analysis, high-purity N_2_ (99.999%) was used. Approximately 5 mg of the previously homogenized material was weighed into Al_2_O_3_ crucibles without lids. The heating rate of 10°C/min was applied. Parallel measurements were run, and these showed excellent reproducibility (differences between mass change steps <2%).

## Results and discussion

### Thermogravimetric analysis

The thermal degradation of lignin is a complex process because the materials have many components with different decomposition pathways, including competitive and/or consecutive reactions.

In the pyrolysis/carbonization process of AGs between 50°C and 950°C, three stages can be distinctly distinguished, which are very similar to those of the pyrolysis of lignin or resorcinol AGs: the first step begins from the start to 150°C, with a weight loss of about 5% corresponding to the evaporation of water and residual organic precursors. The second step consists in the main smooth degradation between 200°C and 500°C, with a weight loss of 50% centered around 350°C. At this stage, the evolution of a high amount of gases also means the occurrence of substantial chemical changes. The third step after 500°C is related to the slow carbonization with a highly insignificant evolution of volatiles and with almost no mass loss up to 950°C. Three similar stages have been proposed earlier, except the third one, char formation, which is considered to start at above 450°C. At that stage, the recombination and coupling reactions can occur within the particles and the condensation reaction of degradation products takes place (Yu et al., 2021).

The first step was to study the different lignin types. The TGA and derivative thermogravimetric DTG result with AGs prepared using different types of lignin is shown in Supplementary Figure S1. The amount of lignin was, in all cases, 75% of the aromatic part of the AG. There are quite different degradation maxima (320°C for AG-HL-75, 381°C for AG-EOL-pine, and 388°C for AG-DOL-pine), suggesting different degradation pathways. AGs from AG lignins exhibit close maximum degradation temperatures, which are almost 65°C higher than in the case of hydrolysis lignin. The final product forming 25–45 wt% of the initial weight is carbonaceous material (25% for AG-EOL-pine, 35% for AG-DOL-pine, and 45% for AG-HL-75). Here, the effect of different origins of lignin is seen.

**FIGURE 2:**
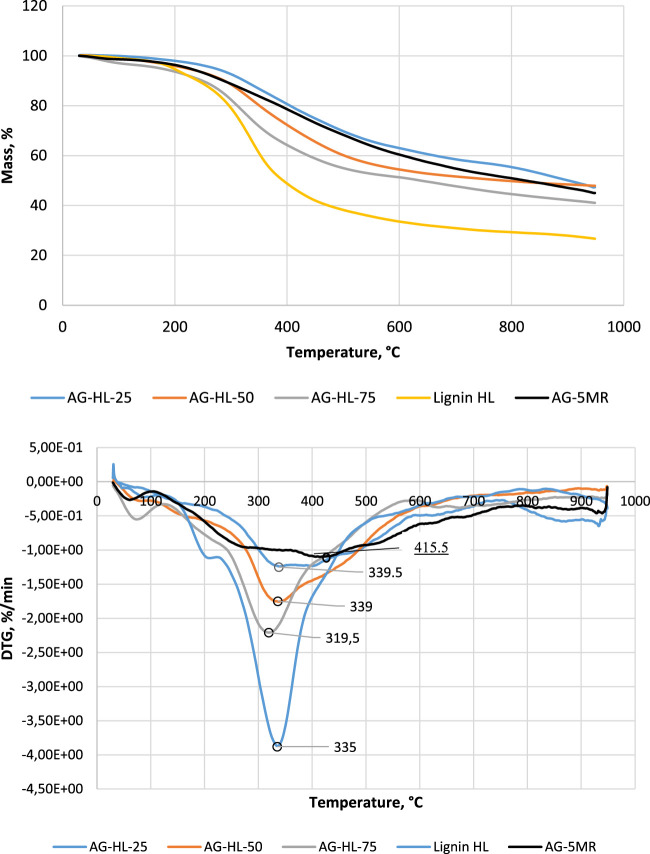
TGA and DTG of AG samples with different contents of hydrolysis lignin. For comparison, data on pure lignin (lignin-HL) and 5-methylresorcinol–formaldehyde aerogel (AG-5-MR-FA) have been added.

Further studies were carried out on aerogels with different lignin contents—25%, 50%, and 75%. The lignin used was birch hydrolysis lignin. The experiments show that the lignin content can be increased to 80% to obtain a stable composite aerogel. Figure 2 shows the thermal analysis data for AGs with different lignin contents. The TGA curves show a fairly general degradation characteristic of resorcinol–formaldehyde AGs (Maldonado-Hodar et al., 1999).

The differences are more obvious on the DTG curves. The difference is in the weight loss peak temperature, which is 415°C for AG-5-MR-FA and in the range of 320°C–339°C for lignin-based AGs, depending on the content of lignin. From the DTG curve, one can follow two degradation processes: one is related to the degradation of lignin structure units (the 320°C–339°C range), and the other is related to the resorcinol structure degradation (above 425°C). The lower temperature of degradation maxima points to the weaker bonds in the lignin in the AG matrix. Virtual DTG curve decomposition could give an idea of the thermal reactions during the pyrolysis of the investigated materials (figures in the Supplementary Material). Here, the simplest approach with Gaussian curves was performed by varying the peak temperature, peak height, and peak number to match the measured DTG. The results are presented in the Supplementary Material. Two different ranges are seen in the reactions related to the degradation of the material—for lignin with the tip temperature in the range of 330–335°C and degradation of 5-MR part in the range of 425°C–430°C. However, the decomposition of the pure 5-MR-FA aerogel DTG curve gives a much more complicated picture of the thermal degradation of the resorcinol–formaldehyde material. For pure lignin degradation, this virtual decomposition also follows the following three main stages: low-temperature range of 200°C–280°C with two peaks, intensive degradation with the maximum at 338°C, and carbonization at higher temperatures >600°C.

The higher mass of the residual for AG-5-MR-FA compared to lignin AGs indicates that the content of the volatiles in lignin is higher compared to that in 5-MR. At above 600°C, the decarbonylation of alkyl side chains takes place along with a substantial reduction in the functional groups and the aromatic condensation reactions occurring to cause the aromatic polycondensation, leading to the formation of amorphous carbon (Brebu and Vasile, 2010). These volatiles participate in the char formation reactions at higher temperatures. This reaction fills the porous carbon structures from the resorcinol–formaldehyde carbonization, resulting in an amorphous-like non-porous structure and a very dense carbonaceous material. The pyrolysis process in terms of weight loss is very similar for all samples, which provides evidence of the similarity of the process for resorcinol-based and lignin-based materials.

### FTIR analysis

**FIGURE 3:**
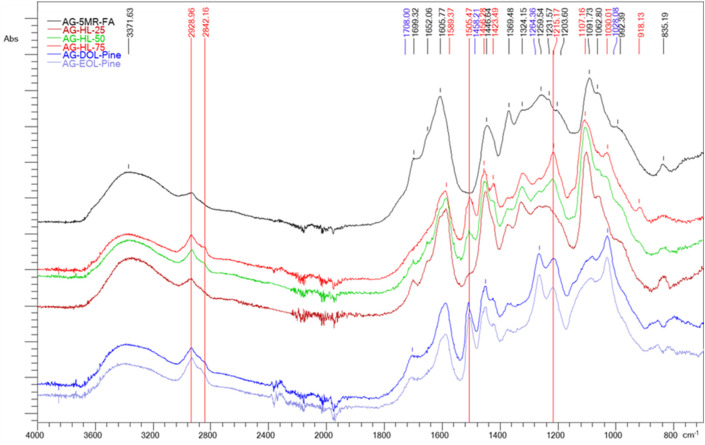
ATR-FTIR spectra of lignin-based and 5-MR-FA aerogels.

Fourier-transform infrared spectroscopy (FTIR) analysis shows the main differences in the structure of AG-5-MR-FA and lignin-based AGs (Figure 3). In the FTIR spectra of the lignin-based AGs, a peak at 1,505.47 cm^–1^ (stretching vibrations in the aromatic structure C=C) and peaks at 2,842.16 cm^–1^ and 2,928.96 cm^–1^ (symmetric and asymmetric stretching vibrations of C–H related to methyl and methylene) are observed, while their intensity is increased in the spectra of AGs, which have a higher content of lignin. The same tendency was observed for the phenolic hydroxyl groups (1,215.17 cm^–1^).

### Raman analysis

Raman spectroscopy of the materials studied provides some additional information, especially on CAs. The spectra of the CA show two broad and strongly overlapping peaks with intensity maxima at 1,336 cm^-1^ (D-band) and 1,593 cm^-1^ (G-band) and with similar intensities. This structure and the Raman spectra of the CA can be interpreted as highly disordered graphitic structures. The G-band is always present in all carbon and graphitic materials, and the D-band is associated with imperfections or loss of hexagonal symmetry in the carbon structure (Gunko et al., 2013). The spectra of the AGs are not very characteristic, and it is difficult to draw serious conclusions about the structure (figure in the Supplementary Material).

### Morphology of aerogels

S_BET_ and porosity were determined by the N_2_ adsorption/desorption technique, which allows the calculation of average pore sizes and the distribution, as well as the S_BET_ of the material. Data on the porosity of AGs, both organic and carbon, are presented in Table 2. No difference was found in the carbon samples prepared using different temperature programs.

Hydrolysis lignin-based organic AGs show a relatively high S_BET_, which is close to that of AG-5-MR-FA. AG lignin-based organic AGs (AG-EOL-pine and AG-DOL-pine) have an even higher S_BET_ compared to that of AG-5-MR-FA. The differences in CAs are more evident, especially in the case of CA lignins, which is indicative of the highly different behavior of the materials during the carbonization process. The organic AGs from hydrolysis lignin do not exhibit a very high S_BET_, and the decrease in S_BET_ during carbonization is not very high either. The reason for this can be related to pore formation during carbonization—the total pore volume is substantially lower, and the pore diameters are smaller. The resulting material is harder and denser compared to the CA derived from 5-MR-FA. Therefore, it can be observed that S_BET_ decreases with a higher HL content. However, there is a significant difference between EOL-pine- and DOL-pine-based CAs. After the pyrolysis procedure, the S_BET_ value of the former is more than 15 times higher, while the total pore volume is much lower.

**FIGURE 4:**
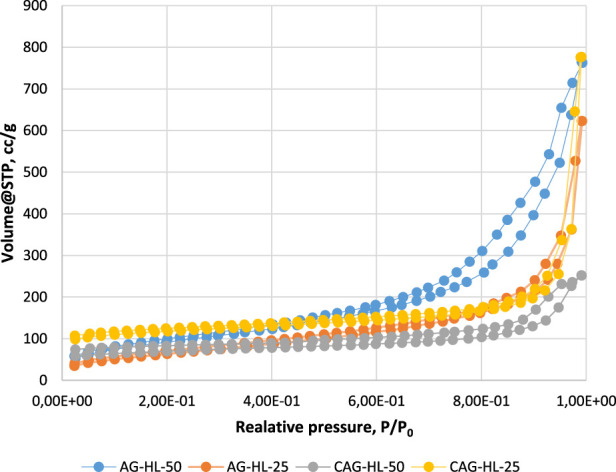
N_2_ absorption curves for lignin-containing AGs and related CAs.

The N_2_ adsorption–desorption isotherms of the prepared AGs (lignin-based, in which 25% and 50% of the 5-MR were replaced by lignin and related CAs, respectively) are shown in Figure 4. The Supplementary Material presents the pore size distribution for these aerogels (Supplementary Figures S13, S14).

According to the IUPAC classification, the obtained N_2_ adsorption/desorption isotherms for AGs are characteristic of mesoporous adsorbents, where the adsorption hysteresis is clearly seen and can provide information about the texture (e.g., pore size distribution, pore geometry, and connectivity) of the mesoporous material.

The initial region of the isotherms at low P/P_0_, which indicates the presence of micropores in addition to mesopores in the gel structure, is very similar in the case of all the samples and is not characteristic of these AGs specifically. It is well known that organic resorcinol–FA AGs do not have microporosity in their structure. Hysteresis loops for gels in the P/P_0_ range of 0.2–1.0 reveal that mesoporosity is dominant in their structures.

From a previous study, it became clear that AG-5-MR-FA exhibits a network structure composed of agglomerates of uniform spherical particles (Peikolainen et al., 2012). The addition of lignin into the structure of AG tends to induce some disordering, leading to a partial opening of the network structure. This observation can also be extended to CAs.

### SEM analysis

SEM pictures of organic AGs confirm the similarity between AGs having a uniform structured tenuous network of clustered nanopores of sizes approximately 10–30 nm and CAs having those of approximately 3–18 nm.

As demonstrated by 31P-NMR quantitative analysis in a previous publication by Jõul et al. (2022), a significantly higher amount of aromatic OH groups was found after the dioxane AG extraction than when using ethanol. This did not significantly affect the properties of organic AGs (Supplementary Figure S2), but if these organic AGs are compared with CAs (Supplementary Figure S3), it can be said that differences in the morphology of CA-DOL-pine and CA-EOL-pine are caused by changes in the molecular structure of AGs during the pyrolysis procedure.

Replacing 25% of 5-MR with lignin creates light and porous CAs, while AG prepared from materials with 50% of lignin content already gives a solid and dense monolith and that with 75% gives a hard solid monolith with needle crystals on the surface (Supplementary Figures S4, S5).

For comparison, Supplementary Figure S6 shows AG-5-MR-FA and its carbonized form CA-5-MR-FA, which is a light and fragile CA.

Based on the previous knowledge of CA-5-MR-FA, it can be expected that replacing 5-MR with lignin creates AGs with adjustable S_BET_ and a certain amount of micro-, macro-, and meso-pores and also enables easy structural modification with other elements (N and S) and metals.

## Conclusion

The present study shows that the extraction processes of the pine AG lignin and the birch hydrolysis lignin generated the material with a high reactivity to form a gel with FA. It was possible to produce FA AGs from lignin with the addition of 5-MR (25%–75%). The additional pretreatment of the precursor lignin with NaOH allows increasing its solubility, which, thus, enhances the gelation process. The commercially available hydrolysis lignin is a material that has good reactivity to form gels with 5-MR and FA, yielding monolithic mesoporous AGs with a relatively high S_BET_ of up to 343.4 m^2^/g. Supercritical CO_2_ drying is superior and provides stable AGs. The obtained lignin-based organic AGs were used as the raw material for porous CAs, but the pyrolyzed organic AGs with a high lignin content did not exhibit as outstanding porous properties as 5-MR-FA-based CAs. The search for some kind of lignin pretreatment is needed to guide the lignin pyrolysis toward a monolithic carbonization process.

For exploration of potential future trends in the case of lignin-based AGs, particularly for carbon materials, further investigation of lignin pyrolysis is necessary to get correlations between the lignin structure and the resulting morphology of the carbon material.

## Data Availability

The original contributions presented in the study are included in the article/Supplementary Material; further inquiries can be directed to the corresponding author.
