# Supplementary material for: Preparation and characterization of lignin-derived carbon aerogels
Source: Front Chem. 2024 Jan 8;11:1326454. doi: 10.3389/fchem.2023.1326454 (PMC10801266; doi:10.3389/fchem.2023.1326454)
Supplement: Supplementary file 1 [file DataSheet1.pdf]

Supplementary information

TGA and DTG result with aerogels prepared using a different type of lignins

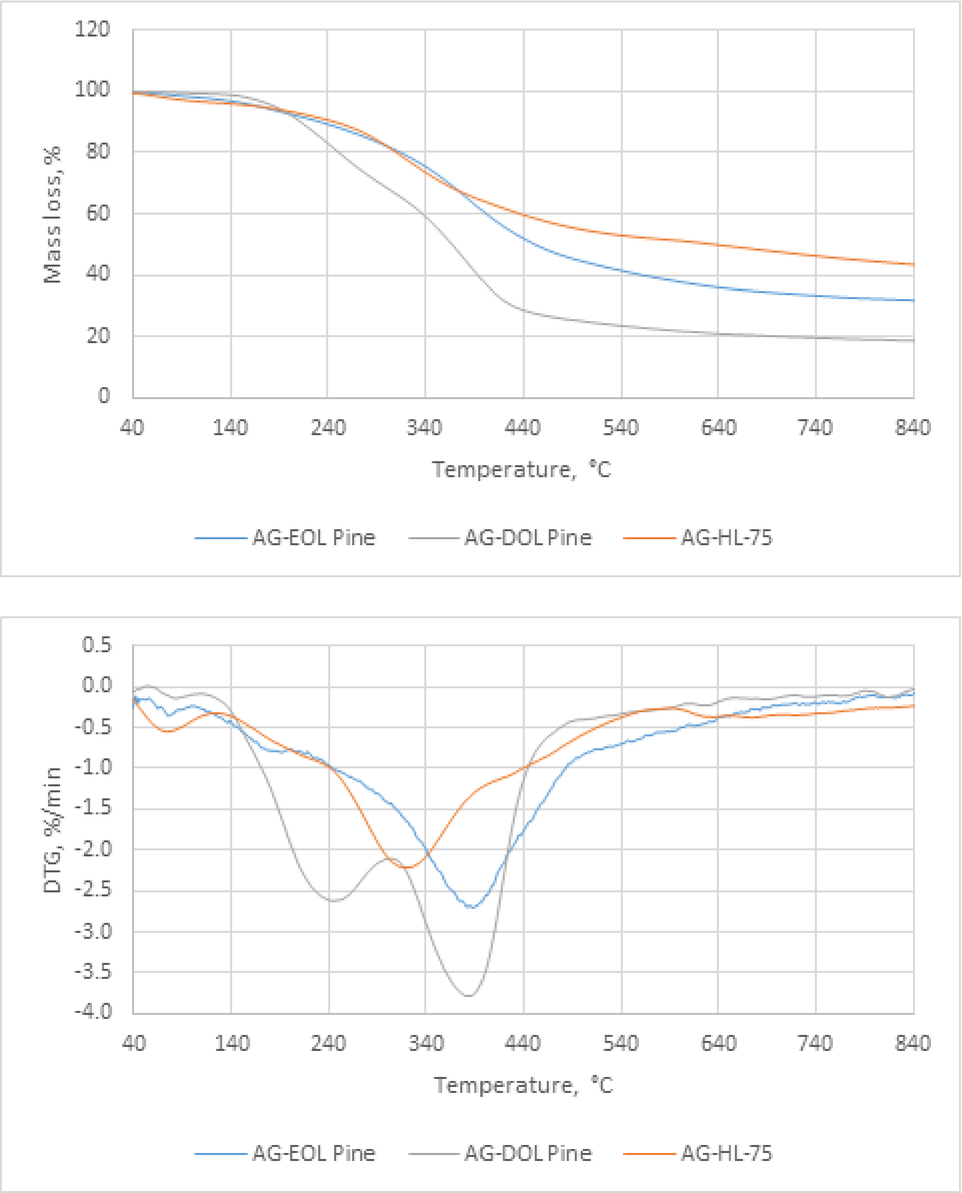

Figure sup1. TGA analysis of lignin containing aerogel samples based on different lignins.

Decomposition of DTG curves

The peak width (DTG at 0 %/min) was about 230 degC. Among the parameters of the peaks, only the peak tip temperatures were varied (the range of values is given in the Table sup 1.), as well as the peak heights. The number of peaks used in the analysis ranged from 8 to 10 (8 for lignin, 9 for AG-HL-75, and 10 for the other samples) and was chosen to ensure that the calculated cumulative DTG curve (black dotted line) matched as closely as possible the measured DTG curve (grey solid line) over the temperature range 100 to 600 degC, while keeping the number of peaks as small as possible.

Each peak used in the calculation of the total DTG curve symbolizes the set of reactions occurring in that peak temperature range. The relative importance of the corresponding reaction sets is given in the attached table. The corresponding values are derived from the heights of the peaks.

Table sup 1. Decomposition parameters.

	Peak temperature, °C								
Sample	150	200-210	267-280	330-335	380-386	425-430	459-470	495-506	570-575
Lignin		0.138	0.087	0.514	0.007	0.145		0.058	0.051
AG-HL-75	0.016	0.127	0.181	0.342	0.040	0.171		0.082	0.040
AG-HL-50	0.050	0.081	0.115	0.266	0.079	0.181	0.046	0.107	0.075
AG-HL-25	0.046	0.058	0.097	0.208	0.121	0.160	0.053	0.150	0.107
AG-5MR	0.032	0.093	0.154	0.148	0.111	0.146	0.032	0.153	0.130

Figure sup 2. Ligniin

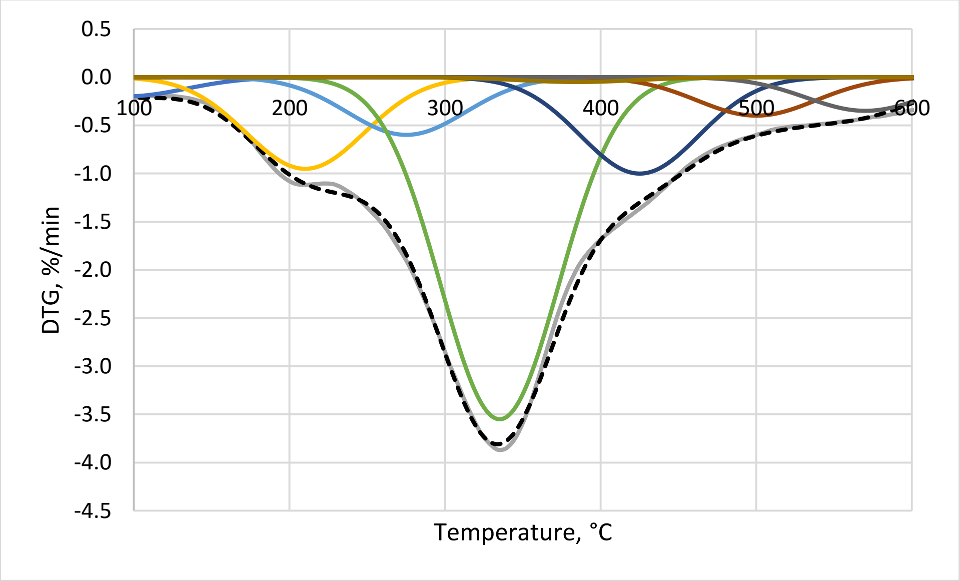

Figure sup 3. AG-HL-75

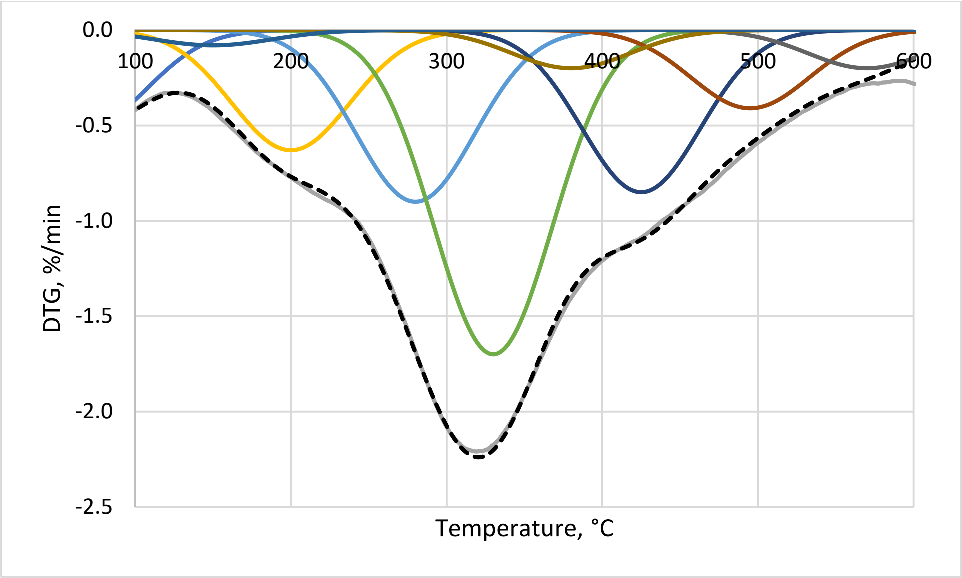

Figure sup 4. AG-HL-50

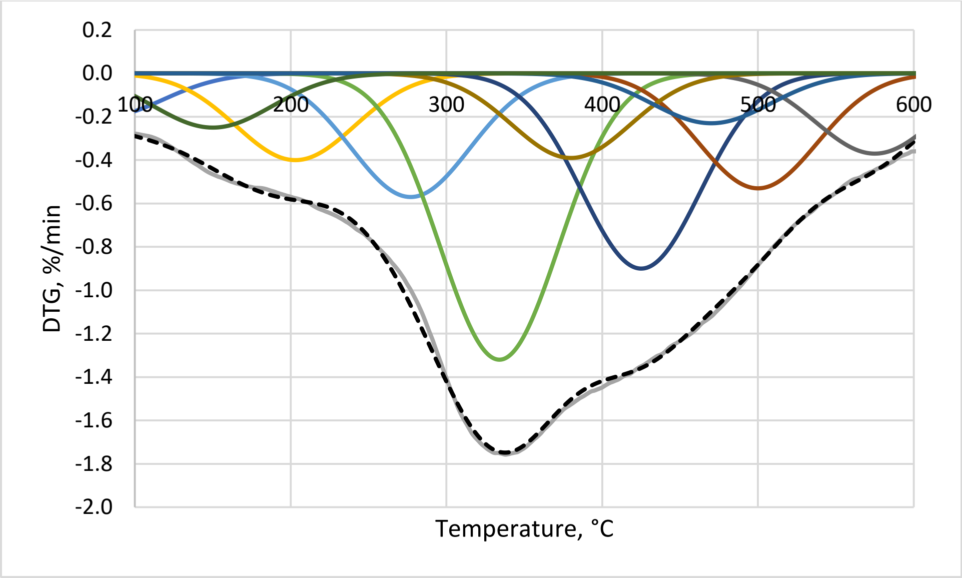

Figure sup 5. AG-HL-25

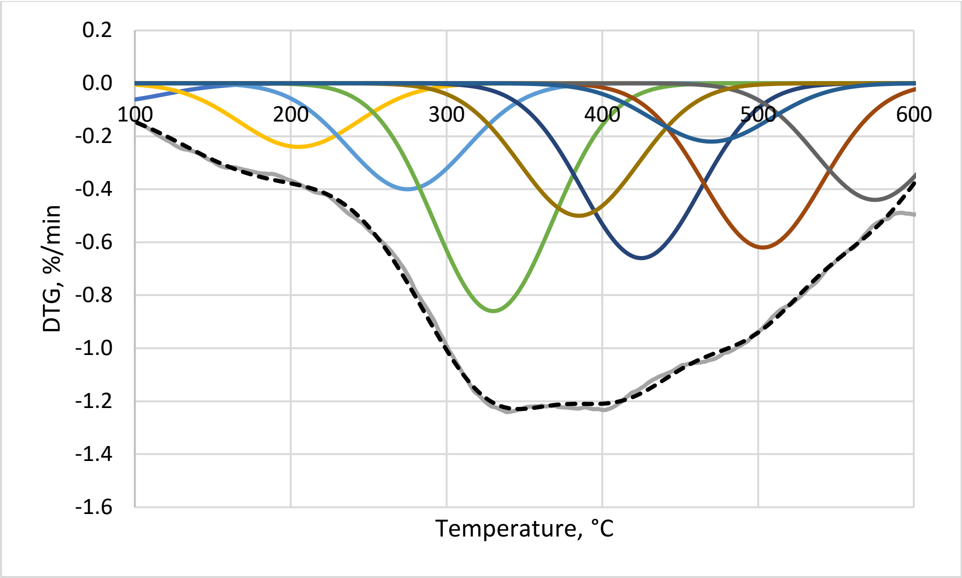

Figure sup 6. AG-5MR

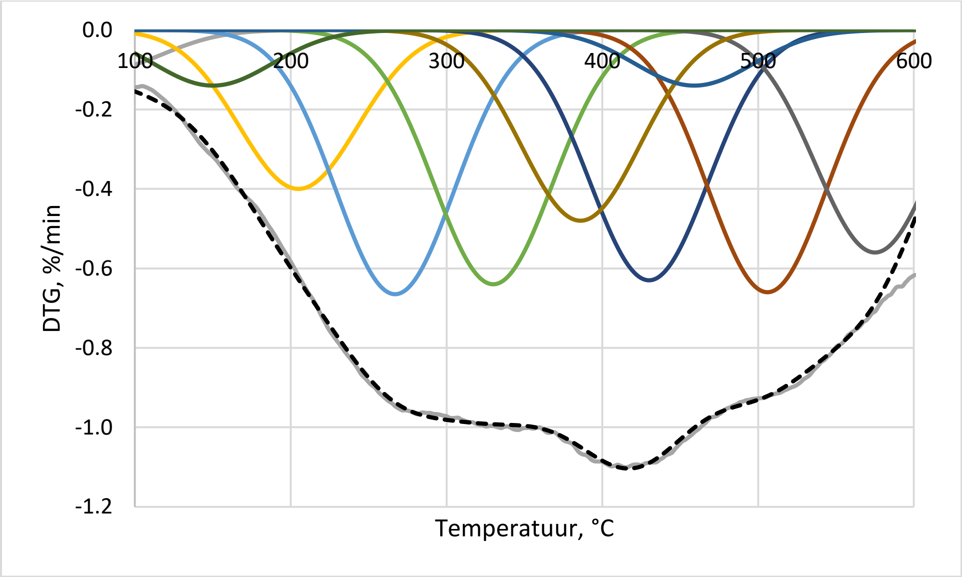

Raman spectra of the samples

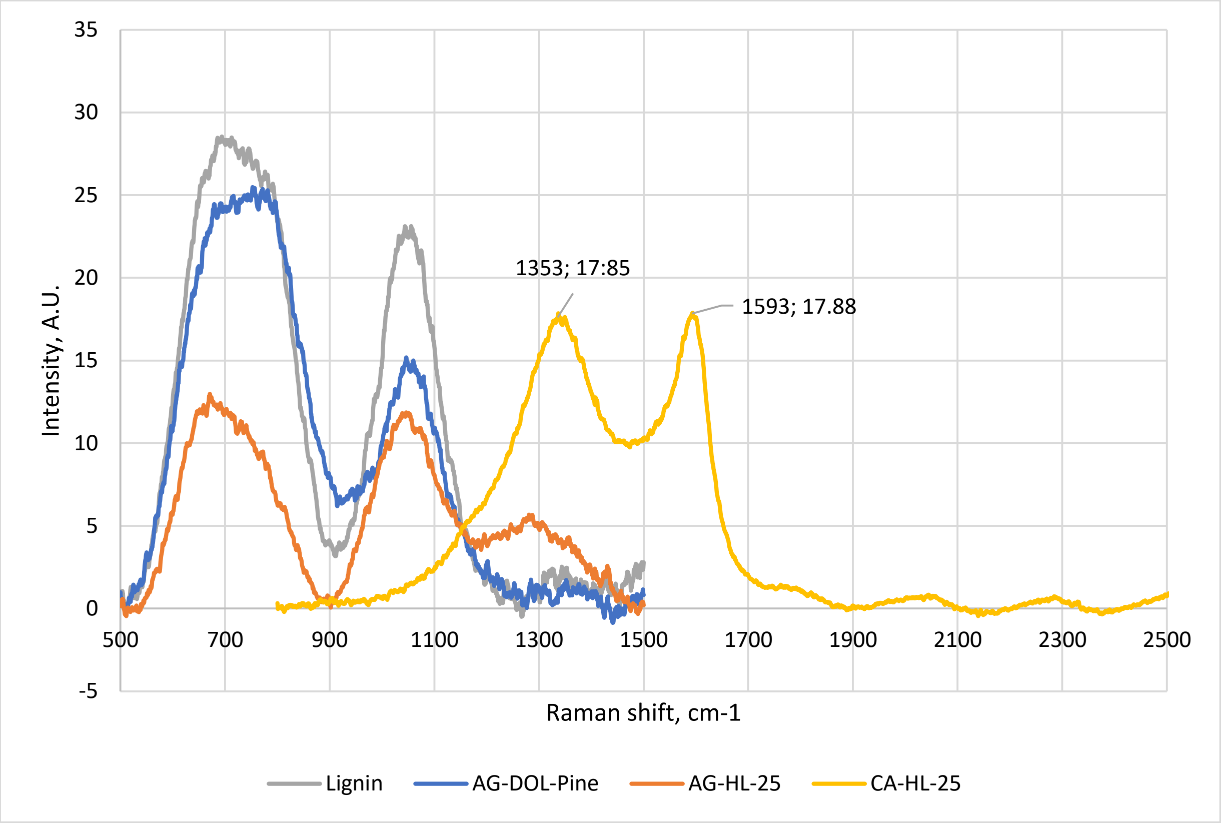

Figure sup 7. Raman spectra of different aerogel samples (organic aerogels with different lignins – AG-DOL-pine and AG-HL-25; carbon aerogel CA-HL-25, and for comparison pure hydrolysis lignin).

Morphology of samples.

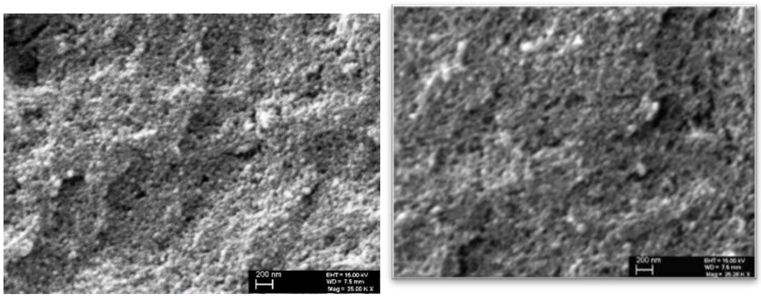

AG-DOL-Pine AG-EOL-Pine

Figure sup 8. SEM pictures of aerogel from organosolv lignins

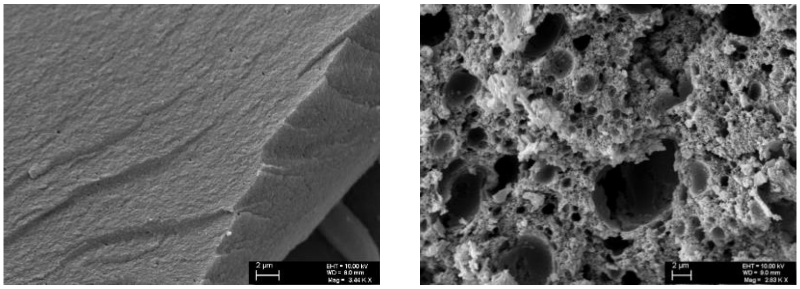

CA-DOL-Pine CA-EOL-Pine

Figure sup 9. SEM pictures of carbon aerogel from organosolv lignins

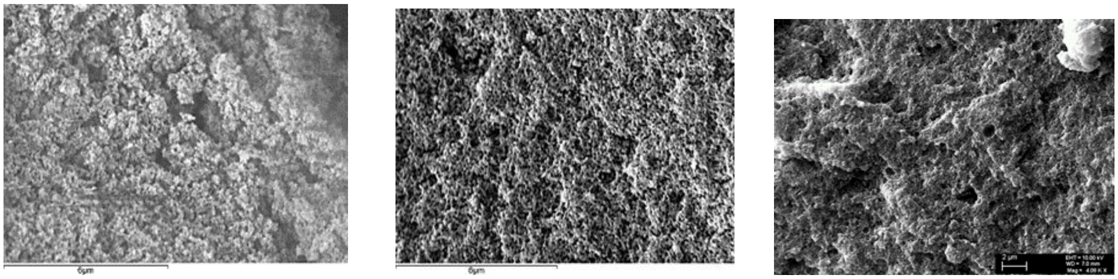

AG-HL-25 AG-HL-50 AG-HL-75

Figure sup 10. SEM pictures of aerogel from different content of hydrolysis lignin

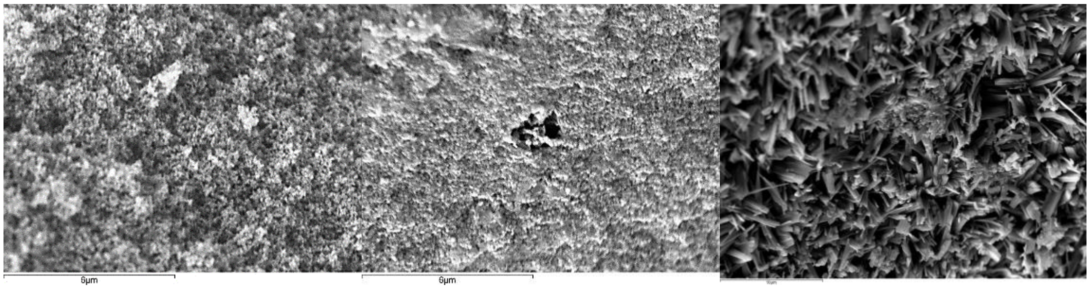

CA-HL-25 CA-HL-50 CA-HL-75

Figure sup 11. SEM pictures of carbon aerogels from different content of hydrolysis lignin

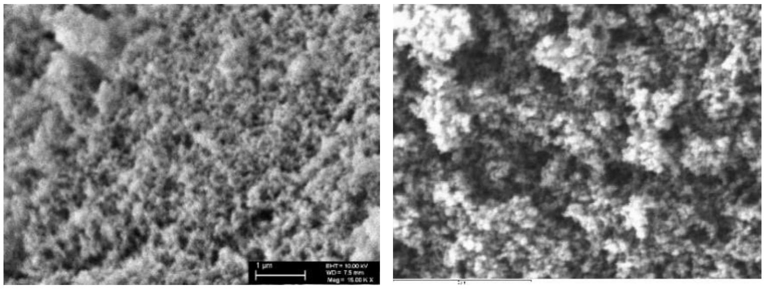

AG-5-MR-FA CA-5-MR-FA

Figure sup 12. SEM pictures of organic and carbon aerogel from pure 5-MR-FA

Aerogels pore size distribution

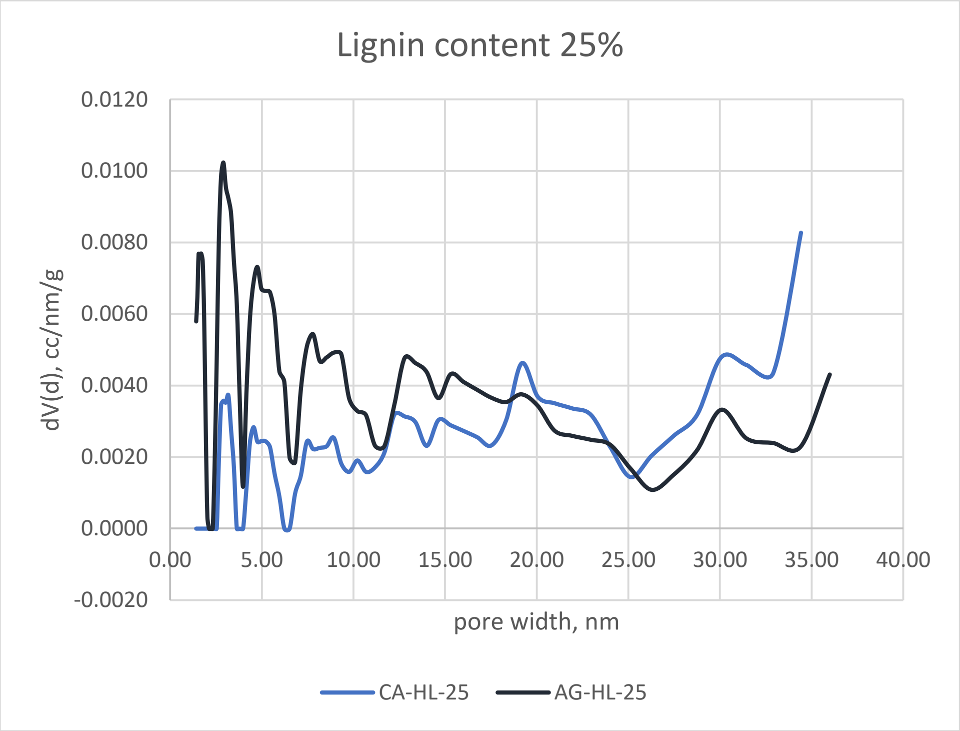

Figure sup 13. Pore size distribution for aerogels with 25% lignin content.

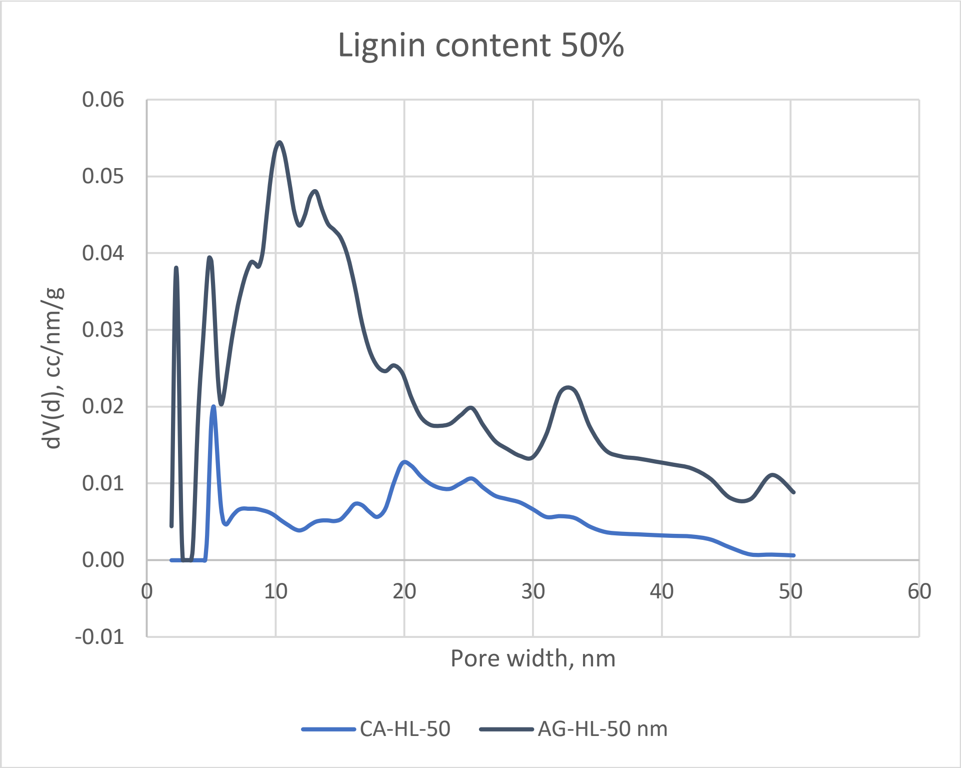

Figure sup 14. Pore size distribution for aerogels with 50% lignin content.
